# Activity-dependent memory organization in the early mammalian olfactory pathway for decorrelation, noise reduction, and sparseness-enhancement

**DOI:** 10.1186/1471-2202-12-S1-P186

**Published:** 2011-07-18

**Authors:** Benjamin Auffarth

**Affiliations:** 1 Computational Biology and Neurocomputing (CBN), Royal Institute of Technology, 100 44 Stockholm, Sweden; 2 Stockholm Brain Institute, Karolinska Institute, 171 77 Stockholm, Sweden

## 

Animals are able to distinguish a large number of different odors (Axel, 1995) and this is crucial in social interaction, feeding, and mating. This discriminatory performance is due to a series of information processing steps at several levels of the olfactory system. Epithelial olfactory receptors (ORs), expressed on celia of olfactory receptor neurons (ORNs), bind to different odour molecules. Axons of ORNs converge by OR type into neuropil structures in the olfactory bulb (OB), called glomeruli, and pass signals to M/T cells. A striking feature of the olfactory bulb is its plasticity, including formation of connections. [1]

It has been shown [cf. 2] that axonal growth could be guided by activity and we mimic this principle in our abstract computational model of the early mammalian olfactory system. In our model, connections cluster together based on correlated excitability of pre-synaptic neurons, so that artificial glomerular structures self-emerges based on odourant receptor (OR) identity. This is based on a model of cortical processing [3]. We show that activations on the postsynaptic, bulbar layer become sparser and less correlated, principles which König and Krüger [4] postulated to be fundamental to information processing in the brain. By performing vector quantization of responses of ORNs providing input to one such cluster we derive response patterns of the projection neurons, Mitral/Tufted units. We evaluate to what extent this code is capable of distinguishing a large number of different odours. We provide an information theoretic evaluation of encoding, draw parallels to biological systems, and argue for biological plausibility with respect to computational principles and different modeling aspects.

**Figure 1:**
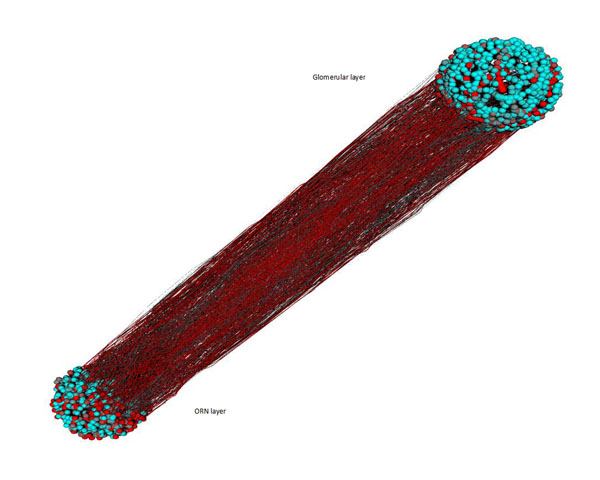


Figure 1 shows a 3D visualization of the ORN layer (bottom), the glomerular layer (top), and axonal projections between the two layers. Neurons are indicated by spheres and connections by cones. Activity to a specific odourant is expressed by graded colour from turquoise to red (figure 2)

**Figure 2:**
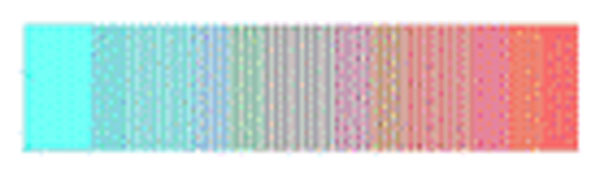


The simulated network consisted of 128 odourant receptor types and 10 ORNs per odourant receptor type.

It can be seen that the axons cluster together on the way to the glomerular layer and that active neurons become more clustered in the glomerular layer with respect to the ORN layer, where activity is fuzzily distributed.

## References

[B1] Lledo Pierre-Marie Gilles Gheusi Jean-Didier Vincent Information Processing in the Mammalian Olfactory System Physiological Reviews 2005 85 1 281 317 10.1152/physrev.00008.2004 15618482

[B2] Ming G Electrical activity modulates growth cone guidance by diffusible factors Neuron 2001 29 2 441 452 10.1016/S0896-6273(01)00217-3 11239434

[B3] Lansner Anders Simon Benjaminsson Christopher Johansson Agustín Gutiérrez & Santiago Marco From ANN to Biomimetic Information Processing 2009 188 Biologically Inspired Signal Processing for Chemical Sensing 33 4319433267

[B4] König Peter Norbert Krüger Symbols as self-emergent entities in an optimization process of feature extraction and predictions Biological Cybernetics 2006 94 4 325 334 10.1007/s00422-006-0050-3 16496197

